# Persistent Mapping of Sensor Data for Medium-Term Autonomy

**DOI:** 10.3390/s22145427

**Published:** 2022-07-20

**Authors:** Kevin Nickels, Jason Gassaway, Matthew Bries, David Anthony, Graham W. Fiorani

**Affiliations:** 1 Department of Engineering Science, Trinity University, San Antonio, TX 78259, USA; 2 Southwest Research Institute, 6220 Culebra Road, San Antonio, TX 78228, USA; jason.gassaway@swri.org (J.G.); david.anthony@swri.org (D.A.); 3 RC Mowers USA, 2146 E Deerfield Ave, Suamico, WI 54173, USA; mdbries@crimson.ua.edu; 4 US Army CCDC Ground Vehicle Systems Center, 6305 E Eleven Mile Rd, Warren, MI 48092, USA; graham.w.fiorani.civ@army.mil

**Keywords:** robotic mapping, localization, SLAM, optimization, GPS-denied mapping

## Abstract

For vehicles to operate in unmapped areas with some degree of autonomy, it would be useful to aggregate and store processed sensor data so that it can be used later. In this paper, a tool that records and optimizes the placement of costmap data on a persistent map is presented. The optimization takes several factors into account, including local vehicle odometry, GPS signals when available, local map consistency, deformation of map regions, and proprioceptive GPS offset error. Results illustrating the creation of maps from previously unseen regions (a 100 m × 880 m test track and a 1.2 km dirt trail) are presented, with and without GPS signals available during the creation of the maps. Finally, two examples of the use of these maps are given. First, a path is planned along roads that have been seen exactly once during the mapping phase. Secondly, the map is used for vehicle localization in the absence of GPS signals.

## 1. Introduction

Navigation algorithms for autonomous vehicles have been evolving in recent decades. Two major approaches that have been taken are reactive systems and explicit mapping. As systems advance in the SAE autonomy levels [1], they must develop better situational awareness and not only be able to react, but to determine when the navigation system can and cannot safely drive.

One current example of a reactive system that does not explicitly use mapping is Tesla’s AutoPilot [2], which observes the local environment and reacts to it moment to moment. This system reacts to stimuli inside the current sensor range. In other systems, such as Waymo’s Driver-less car [3], a highly detailed map of the immediate environment is pre-processed, often with much human intervention  [4].

The persistent map is a set of data structures and algorithms used to accumulate, optimize, and store processed sensor data. This allows an autonomous system to plan on previously seen, but otherwise unmapped (in the sense of human curation of data about the environment), regions. It also allows localization with respect to this map, in the case of temporary or permanent loss of an absolute positioning reference such as a GPS.

Automated and autonomous unmanned ground vehicles (UGVs) commonly rely on Global Navigation Satellite Systems (GNSS) like the Global Positioning System (GPS) to locate themselves in the world and perform path planning, navigation, and platooning. However, GPS is not always available due to geography, nearby structures, or adversarial jamming or spoofing. In the absence of GPS, many current systems use dead reckoning—localization with proprioceptive sensors only—to estimate absolute vehicle pose and position relative to the desired path. Without absolute position measurements, errors in vehicle position build up over time, a well-known phenomenon known as drift [5]. Therefore, one or more supplemental localization systems are used to enable long-term autonomous operation in these environments.

The United States Army’s Combat Capability Development Command Ground Vehicle Systems Center (GVSC) is interested in warrior–machine interfaces and autonomous navigation, including in GPS denied environments. To cite a few examples of programs that GVSC has collaborated on, Kania et al. [6] describe a program, the Dismounted Soldier Autonomy Tools (DSAT) that enable warrior–machine interfaces and vehicle architectures on combat-relevant platforms, including localization designed to be robust to GPS drop-outs. Schaefer et al. describe an evolution of this system as part of a program called Wingman, which “focuses on human–machine collaboration, decision-making, human–machine combat training, and advanced manned-unmanned teaming operations” [7]. Towler et al. [8] describe a further evolution and integration of the Robotic Technology Kernel, with an eye toward code re-use and security in the military environment.

The persistent map described in this paper was originally developed for the MUER program and remains of interest for many other Army and DoD programs. One conceptual scenario that this package enables is route retraverse, where a consistent traversability map is generated as the vehicle moves around in a previously unmapped environment. At some later point, this map is called upon to serve as a basis for planning, for example, egress from the area. We define retraverse as traveling over an area that has been traversed, to distinguish the task from retero-traverse [9], which we define as replaying a previously traversed route, also known as teach and repeat [10].

The persistent map is built around the concept of deforming continuous manifolds from odometry space into a common global map. This deformation is continuously optimized to maintain local consistency while optimizing several important metrics with respect to whatever local and global information is available. Multiple distinct layers of map data can be maintained, including estimated cost of traversal, minimum distance to observation, altitude, and uncertainty.

First, related work in localization and mapping is described in Section 2. The discussion of the persistent map begins in Section 3 with an overview of the map’s representation, terms, and geometric concepts. The optimization approach itself is discussed in Section 4, with costs that allow the balancing of correlation of overlapping source sheets, incorporation of available GPS information, and amount of deformation of the map structures themselves being described in detail in Section 4.1. Section 5 details the approach as a step-by-step algorithm. Finally, some representative results from utilizing the persistent map for planning in a previously explored but unmapped area are given in Section 6.

The primary contribution of this article is a case study in organizing and processing costmap data in such a way as to be revisable as new data (processed sensor data or GPS fixes) is observed, but usable at any point for autonomous tasks such as planning on the map or retraversing a previously observed area.

## 2. Related Work

The process of building a map of the environment, while localizing the vehicle within that map, is referred to as Simultaneous Localization and Mapping (SLAM) or sometimes Concurrent Mapping and Localization (CML), and has been studied extensively for several decades. An excellent tutorial is given in [11,12], and a more updated review in [13].

Three main paradigms for the SLAM problem have emerged. In Extended Kalman Filter based approaches (EKF-SLAM), the locations of features relative to the robot in the environment are each tracked with an EKF, with careful attention to the covariances of the location of the robot with respect to the map and the features with respect to the robot.

Another approach utilizes Rao-Blackwellized Particle Filters, with their basis in Monte Carlo Sampling of possible robot locations, to predict the location of features in the environment based on a robot location. Then, particles for whom the expected and observed locations match well are strengthened and particles for whom the locations do not match are weakened. Over time, the robot locations that best match the actual robot location gain strength and are replicated and those who do not are pruned. The most well-known implementation of Rao-Blackwellized Particle Filters for SLAM is probably that given in the Gmapping algorithm [14] distributed with ROS [15].

The third class of SLAM algorithms constructs a graph where nodes correspond to robot locations and edges correspond to constraints on those locations, either from location to location constraints (i.e., odometry) or feature to location constraints (i.e., feature measurement) [16]. This graph is adjusted by treating every constraint as slightly flexible and batch optimizing the entire graph. One of the most famous graph-SLAM approaches is Google Cartographer [17], and a recent implementation is given in [18].

Recent SLAM advances such as CT-ICP [19] perform better on fast moving vehicle platforms by compensating for noise in the lidar point cloud induced by motion. Our method operates on costmaps or 2D projections of point clouds, allowing us to complement these methods and provide a robust, long term mapping solution.

Many deep learning methods have been proposed that use deep neural networks to create robust feature detectors that outperform feature detectors from classical computer vision. While these feature detectors can significantly improve the initial stages of a SLAM pipeline, they rely on foreknowledge of the environment, frequently require dedicated hardware in the form of machine learning accelerators and GPUs, and are typically specialized and trained for a particular environment. For example, Tinchev et al. [20] use labelled point clouds to train a deep neural network that improves point LiDAR segmentation performance. Cattaneo et al. [21] use deep learning of LiDAR point clouds to detect loop closures, which can significantly improve SLAM performance. Gridseth, et al. [22] leverage deep learned features to execute visual teach and repeat in widely varying environmental conditions.

In contrast, our system performs extremely fast mapping and registration on consumer grade CPUs and is robust to a wide variety of operating conditions. We have utilized this system in off-road, on-road, and urban environments without needing to re-train a neural network or modify hyperparameters to achieve acceptable performance.

Within the context of ground and air vehicle robotic localization, exteroceptive robot-mounted sensors such as LiDAR, RADAR, cameras, and sonar are often augmented and combined with proprioceptive sensors such as wheel odometers, gyroscopes, and steering angle measurement, as well as GNSS (Global navigation satellite systems) to arrive at a more robust and accurate location for the robot [23].

The staid Kalman Filter (KF) has been the most common method for integration [24], spawning many variations for special cases. To cite a few recent examples, Woo et al. [25] pose a fuzzy-innovation-based adaptive KF that adaptively updates the measurement covariance to model GPS quality. Abdelaziz et al. [26] study the use of a loosely-coupled extended KF to robustly handle GPS dropouts for extended periods of time, relying instead on the LiDAR data for those periods. Hosseinyalamdary [27] proposes a “Deep KF” which adds a modeling step to the classical prediction and update steps of the KF.

The use of processed sensor data rather than raw LiDAR or visual data in KFs is common, to improve the robustness of the extracted features. For example, Oh et al. [28] use a feature extractor based on a deep variational autoencoder, then use sequences of these features over time to aid the KF. Dong et al. [29] attack the other half of the equation, processing the GNSS signal to extract information about the GNSS carrier phase to improve the fused localization. Mostafa et al.  [30] incorporate both Radar Odometry and Visual Odometry into an integrated system before incorporating GPS information when available to localize a unmanned air vehicle.

As mentioned above, GVSC has been developing Robotics Technology Kernel (RTK), “the Army’s library of modular software packages for ground autonomy [31]”, for over two decades. Flannigan et al. [32] describe one of the earliest programs that led to RTK, the Small Unit Mobility Enhancement Technology (SUMET) program, which investigated a range of Electro-optical imagery for object/region segmentation and classification. Kania et al. [6] describe the Dismounted Soldier Autonomy project, an early foray building on SUMET into teamwork between warfighter and autonomous vehicle. Johnson et al. [33] present an earlier version of the Persistent Map and its interface to digital elevation maps. Seegmiller et al. [34] present an efficient planner used in the RTK toolkit, the Maverick planner. Some recent work showcasing the evolution and use of this library include [35] which describes adaptations to the toolkit for a small low-speed electric vehicle operating in established infrastructure, [36] which uses the toolkit to compete in the “Intelligent Ground Vehicle Competition Self-Driving Competition” in on-road driving, and [37], which describes RTK’s use in a small unmanned indoor ground vehicle capable of site exploitation and radiation sensing.

RTK makes extensive use of processed exteroceptive sensor data, called costmaps, as one of the primary inputs. Several GVSC and SwRI projects have worked extensively on robustly identifying roads, traversable areas, and lethal obstacles in sensor data, and developing a costmap. This prior work means that the use of costmaps for optimization, as opposed to raw sensor data, will be more robust.

## 3. Map Geometry

The persistent map is composed of a set of contiguous sheets of map data. A *sheet* is defined to be the accumulation of locally consistent sensor data, referenced to the odometric coordinate frame [38]. These sheets (called manifolds in [39]) are maintained such that there are no loops or re-traversals within a single sheet. A new sheet is created if the vehicle changes directions, or if for some other reason it is detected that the vehicle is operating in a totally unmapped area, such as just after a freshly loaded map or a map reset. A sheet is intended to be small enough to be locally consistent, similar to the submaps of Google Cartographer [17]. We define the active sheet to be the one that is currently being extended as the vehicle moves.

A sheet is composed of tiles, which are uniformly sized adjacent patches of map data. Each tile is anchored by four vertices, which have both odometric and earth-centered coordinates. As described in [33], this method of defining a map has several advantages over other methods such as pose optimization [17]. In order to use the persistent map for autonomous mission planning, such as retraverse, local consistency is very important. Artifacts such as tearing and gapping will cause the map to become unusable very quickly for autonomous planning of medium term missions.

While the sheets and source tiles are defined in odometric space, we also have a data structure defined in an earth-centered coordinate system—we use WGS84 as our earth-centered coordinate system [40]. Each tile is of uniform size, and the tiling is designed such that there are an integer number of tiles on each latitudinal ring.

The deformation between source sheets and a single output layer specified in earth-centered coordinate systems is continuously updated by the numerical optimization described in Section 4, and is recomputed as needed for visualization and planning.

**Figure 1:**
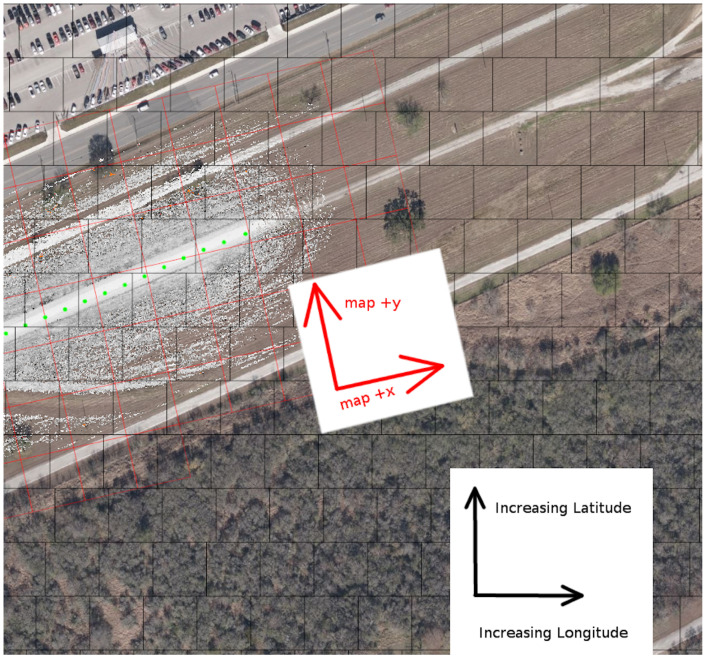
A visualization in mapviz [41] of the costmap layer (the greyscale dots) of a persistent map of a portion of the SwRI test track. Source Tiles are outlined in red, and are aligned to the map space (appearing at an angle in this image). GPS fixes are shown in green. WGS84 Tiles are outlined in black, aligned with longitude/latitude space. All tiles are 128 × 128 quadrangles, at a scale of about 25 m on a side under this vehicle’s sensor configuration. The satellite imagery is shown only for context.

An example persistent map, with background satellite imagery shown for context, is shown in Figure 1.

## 4. Deformation Model

The deformation between source sheets (tracked in odometry space) and the output layer (tracked in WGS84 space) is continuously computed by a numeric optimization [42]. The cost functions used in the optimization penalize non-rigidity, re-projection error between GPS samples and the corresponding location of the GPS antenna in relative coordinates, and image registration error between overlapping regions of the map.

### 4.1. Model Cost Terms

Adjusting the mapping between odometry and global coordinate systems is one of the major tasks of the persistent map. The adjustment is done by defining a cost function and adjusting the map configuration to reduce or minimize the cost function. This approach allows us to consider many influences on the map simultaneously.

The optimization is run continuously as new data are incorporated into the map. Once the vehicle leaves a sheet, no new GPS samples are added to that sheet, but newer sheets are registered against older sheets as they are created and optimized, allowing continual adjustment of previously seen data. As GPS samples may not be reliably available for all areas, or even all sheets, this flexibility to allow older sheets to remain dynamic is important to maintain consistency and accuracy.

There are four types of cost terms used in the optimization: Source Tile Deformation, GPS reprojection, GPS Offset Error, and Costmap Registration.

#### 4.1.1. Source Tile Deformation

The deformation cost minimizes the amount of non-rigid distortion in the map. Distortion accounts for error in the relative odometry but should be limited to prevent kinks or tears in the map. It is expected that odometric error grows linearly over time [5].

Each sheet is composed of source tiles. Each source tile contributes a deformation cost equal to the sum of the squared difference in length of each side of the tile and diagonal between the deformed and undeformed state.

For a rectangular tile with undeformed vertices $v_{0}$, $v_{1}$, $v_{2}$, and $v_{3}$ and deformed vertices $v_{0}^{\prime }$, $v_{1}^{\prime }$, $v_{2}^{\prime }$, and $v_{3}^{\prime }$, the deformation cost term is

(1) $$\begin{array}{cc} J_{\mathrm{deform}}\left(v_{0,1,2,3}\right)= & \left(|\right|v_{1}-v_{0}\left|\right|-\left|\right|v_{1}^{\prime }-v_{0}^{\prime }{\left||\right)}^{2}+\left(|\right|v_{2}-v_{1}\left|\right|-\left|\right|v_{2}^{\prime }-v_{1}^{\prime }{\left||\right)}^{2}+\\ \; & \left(|\right|v_{3}-v_{2}\left|\right|-\left|\right|v_{3}^{\prime }-v_{2}^{\prime }{\left||\right)}^{2}+\left(|\right|v_{0}-v_{3}\left|\right|-\left|\right|v_{0}^{\prime }-v_{3}^{\prime }{\left||\right)}^{2}+\\ \; & \left(|\right|v_{2}-v_{0}\left|\right|-\left|\right|v_{2}^{\prime }-v_{0}^{\prime }{\left||\right)}^{2}+\left(|\right|v_{3}-v_{1}\left|\right|-\left|\right|v_{3}^{\prime }-v_{1}^{\prime }{\left||\right)}^{2} \end{array}$$

#### 4.1.2. GPS Reprojection

The GPS reprojection cost is used to incorporate GPS samples, where they are available, into map deformation. Each time a GPS reading is received, the location of the antenna in the relative coordinate frame is also recorded, and attached to the respective source tile. Let $p_{\mathrm{ant}}$ represent the antenna location in odometric coordinates, g(p) represents the affine transform from the undeformed to the deformed configuration as modified by the source tile vertices $v_{0,1,2,3}$. The deformation cost term associated with GPS samples is thus:

(2) $$\begin{array}{cc} J_{\mathrm{gps}}\left(p_{\mathrm{ant}},e_{\mathrm{offset}},v_{0,1,2,3}\right) & =\Sigma ||p_{\mathrm{ant}}+e_{\mathrm{offset}}-g\left(p_{ant}\right)\left|\right| \end{array}$$

where the summation is over all samples for the tile, and $e_{\mathrm{offset}}$ is the offset associated with this GPS source (see Section 4.1.3). This cost term pulls the tile vertices’ earth-centered positions so that the projected antenna locations align as closely as possible with the GPS measurements.

#### 4.1.3. GPS Offset Error Model

In order to track and model the steady state error that is assumed to exist within GPS samples, and to vary slowly over time due to environmental changes, each GPS source has an error term associated with it:

(3) $$\begin{array}{cc} e_{\mathrm{offset}} & =[e_{\mathrm{east}},e_{\mathrm{north}}] \end{array}$$

(4) $$\begin{array}{cc} J_{\mathrm{gpsoff}} & =|e_{\mathrm{east}}\left|+\right|e_{\mathrm{north}}| \end{array}$$

where the 2D offset is comprised of an easting and northing offset, and  the easting and northing errors appear directly in the optimization residual.

#### 4.1.4. Costmap Registration

The costmap registration term ensures that overlapping regions in two sheets align well with one another. For example, if the same obstacle appears in two costmaps, costmap registration ensures that the obstacle will deform to the same location in both sheets. Other GVSC and SwRI projects [6,32,33,34] have worked extensively on robustly identifying roads, traversable areas, and lethal obstacles in sensor data, and developing a costmap. This work means that the use of costmaps for optimization, as opposed to raw sensor data, will be more robust.

This process is critical to operation where GPS is absent or spotty. In the absence of GPS signals to correct for odometry drift, the deformation model would naturally tend toward a rigid transform and carry the odometry errors into the output map. However, if the map already has data for the same area, registration will allow the system to bias the sheet with missing GPS to align with the sheet with GPS, effectively ‘inheriting’ the GPS signals that were collected in the other sheet. Due to the fact that the optimization is ongoing, this could happen in either order temporally, allowing a later sheet with GPS to re-align an earlier sheet without GPS.

Even when GPS signals are consistently available, they exhibit a steady state error that varies slowly over time due to environmental changes [43]. Since the map persists from day to day, sheets for the same area will be collected with different steady state offsets. In off-road environments with small trails, this effect can be enough to artificially constrict (in the map) a trail that is traversable in the world, as illustrated in [33].

This cost is computed in three phases: (1) finding overlapping map regions, called matches, (2) using phase correlation of these regions to find the optimal offset, and (3) allowing this offset to influence the deformation model.

As the active sheet is extended, the vehicle location in odometric space is recorded at regular intervals. These are attractive match points as they are in the center of the patch of sensor data, so the surrounding regions tend to be rich in data to match. During each update step, one unprocessed match point is selected for matching. A region from each sheet overlapping the match region (in earth-centered coordinate system) is analyzed via phase correlation [44] to determine the best offset.

These match pairs are stored and used to influence the deformation model via the cost function

(5) $$\begin{array}{cc} J_{\mathrm{corr}}\left(p_{a},p_{b}\right) & =\left|\right|g_{a}\left(p_{a}\right)-g_{b}\left(p_{b}\right){\left|\right|}^{2} \end{array}$$

which minimizes the distance between the match points $p_{a}$ and $p_{b}$ in the two sheets, as modified by the homographies $g_{a}$ and $g_{b}$.

This cost biases overlapping sheets toward configurations where their costmap data line up.

### 4.2. Calculating the Joint Residual

Using a CERES-based numeral optimization [42], the costs above are individually weighted and an optimal location in earth-centered coordinates for each vertex and an optimal offset in earth-centered coordinates for each GPS source is computed.

(6) $$\begin{array}{cc} \mathrm{OptimizedCeresInputs}=\underset{\mathrm{CeresInputs}}{arg min}[ & \sum_{\forall \;\mathrm{tile}\;\in \;\mathrm{source}\;\mathrm{tiles}}W_{\mathrm{deform}}*J_{\mathrm{deform}}\left(v_{0,1,2,3}\right)\\ + & \sum_{\forall \;\mathrm{samples}\;\forall \;\mathrm{source}\;\mathrm{tile}}W_{\mathrm{gps}}*J_{\mathrm{gps}}\left(p_{\mathrm{ant}},e_{\mathrm{offset}},v_{0,1,2,3}\right)\\ + & \sum_{\forall \;\mathrm{source}\;\in \;\mathrm{gps}\;\mathrm{sources}}W_{\mathrm{gpsoff}}*J_{\mathrm{gpsoff}}\left(e_{\mathrm{offset}}\right)\\ + & \sum_{\forall \;\mathrm{pair}(a,b)\;\in \;\mathrm{tile}\;\mathrm{matches}}W_{\mathrm{corr}}*J_{\mathrm{corr}}\left(p_{a},p_{b}\right)] \end{array}$$

where CeresInputs comprises a latitude and longitude for each vertex, each vertex comprises a variable latitude and longitude that are optimized, and each offset comprises a variable latitude and longitude offset that are optimized, and the *J* terms are given by (1)–(5).

Compared to strictly enforcing these properties as constraints, this approach allows the map to naturally make use of deformation constraints, GPS as available, and costmap alignment, and provides robustness against matching errors, sensor noise, and GPS outages.

## 5. Algorithm

The persistent map module runs in two parallel threads, sharing data between them. The first thread, with pseudocode given in Algorithm 1, continuously optimizes the vertices of the source tiles according to the metrics given in Section 4, generating an optimized set of latitude and longitude points for each vertex on the active persistent map. The second thread, with pseudocode given in Algorithm 2, accumulates new costmap data onto the active sheet, monitors the odometry for direction change or other events that require sheet maintenance, and interfaces with other modules that may require a flattened version of the persistent map, for example to use for planning.
**Algorithm 1** Optimize Persistent Map.**while** True **do**    Import any changed data for optimization    Run Ceres optimizer [42], based on (6)    Update any changed source tile vertices from optimization**end while**

**Algorithm 2** Map Interface.
**while** True **do**    Import any changed source tile vertices from optimization    **if** Vehicle has entered new source tile **then**        **if** Vehicle has changed direction OR vehicle is in unmapped area **then**           Create new source sheet        **end if**        Create new source tile        Add source tile vertices to optimization per (1)    **end if**    **if** GPS reading received **then**        Add GPS sample to optimization per (2)    **end if**    **if** New costmap is available **then**        Select unprocessed match point        **for** Each source overlapping source tile **do**           Compute offset with costmap via phase correlation           Add offset to optimization per (5)        **end for**    **end if**    **if** Output Layer Requested **then**        **for** Each pixel in requested region **do**           Flatten all source tiles overlapping output pixel (as needed)        **end for**    **end if**    update any changed data for optimization
**end while**



## 6. Results

**Figure 2:**
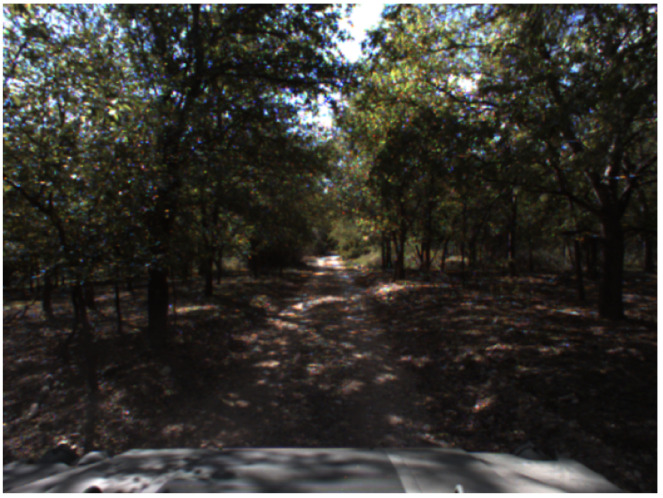
A section of the trail, illustrating overhanging foliage and grass.

This section presents several different scenarios of the creation and use of a persistent map. A vehicle with LiDAR sensors, a GPS receiver, and wheel encoders was manually driven in two scenarios: a test track and an off-road trail. The test track, an oval approximately 100 m by 880 m, is relatively flat, with only occasional trees and a small number of man-made obstacles for landmarks. The trail, shown in Figure 2, is approximately 1.2 km long, with dirt tracks and occasional patches of grass and mud, overhanging foliage, bordered by grass and trees.

### 6.1. Creating a Test Track Map with GPS

**Figure 3:**
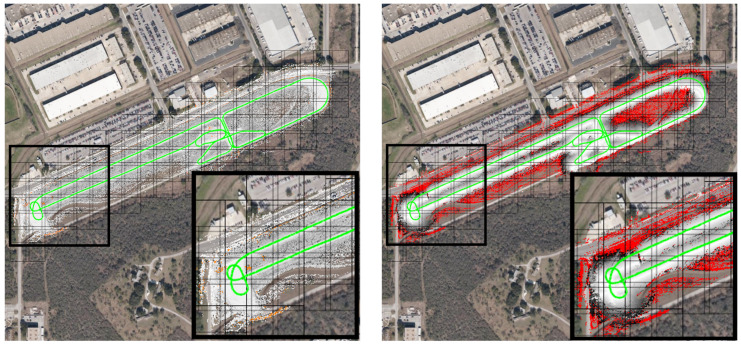
The persistent map (costmap layer on left, sensor distance layer on right) with GPS available through the entire map. GPS tracks are shown in green. Sensor distances over 30 m are shown in red. The insets show a zoomed-in section of the southwest corner of the track. The satellite imagery is shown only for context.

In the first result, a 2.7 km winding path through a test track is driven, resulting in a persistent map with 999 source tiles in nine manifolds, with 6161 GPS samples along the route. The costmap and sensor distance layers of this map are shown in Figure 3.

### 6.2. Creating a Map with Intermittent GPS

This shows the creation of a map with significant GPS dropouts. Due to the optimization, the map tiles without GPS samples are stretched toward those containing GPS samples, resulting in a locally consistent and as globally optimal as possible map. This is preferable, for autonomous vehicles, to maps with tears or gaps in the data.

**Figure 4:**
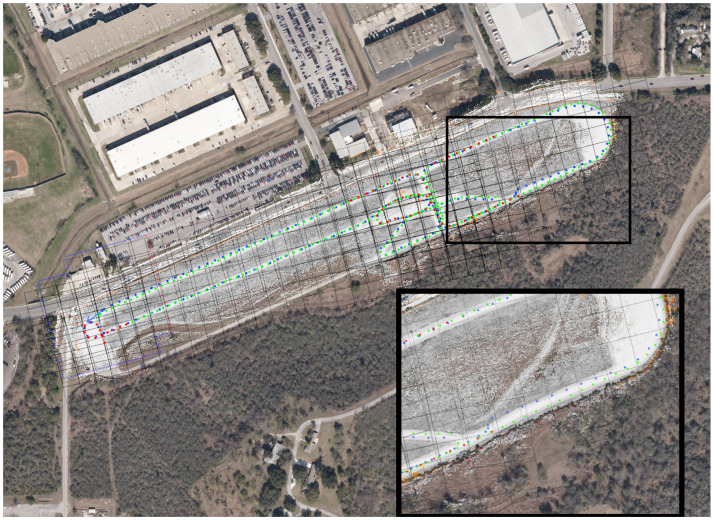
The persistent map (costmap layer) with GPS dropouts throughout the entire map. The available GPS measurements are shown in red, the odometric data in blue. The inset image is a magnified portion of the southwest portion of the left image, showing where GPS was restored as the vehicle traveled west. The satellite imagery and GPS coordinates (green dots) are shown only for context, and are not available to the system.

As described above in Section 4.1, map tiles are registered to one another even if some of them do not have GPS samples associated with them. This allows the system to adjust each manifold if and when those coordinates become available. Figure 4 (inset) shows a section of the map after GPS was restored. Notice the shape of the road in this region—it curves but is traversable on the west, east, and northwest egresses. The optimization has not torn or closed off the road.

**Figure 5:**
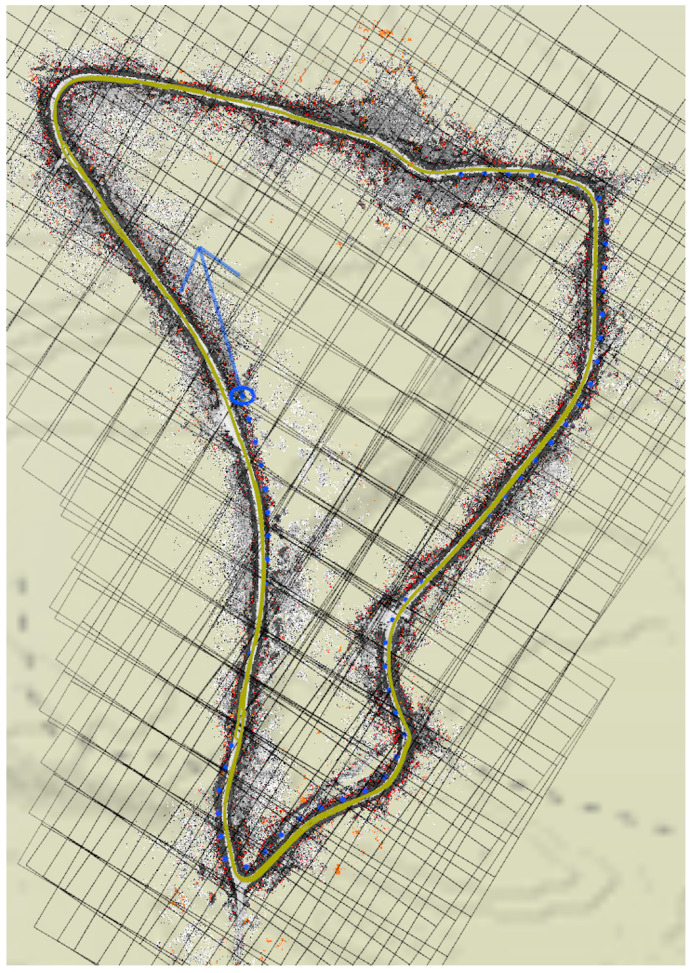
A map of a 1.2 km trail. This was created by one traversal of the loop with GPS, and about one and half more without GPS. The blue arrow represents the estimated location of the vehicle at the point where the map was saved, and the blue dots a location history.

Similarly, a dirt trail of about 1.2 km in length was mapped, as shown in Figure 5, with one loop of GPS-available data plus one and a half more of GPS-denied data.

### 6.3. Localization Based on a Dynamic Persistent Map

If an area has been driven before, with partial or complete GPS coverage, a map can be re-used [45] to help localize the robot in the event of GPS dropout or spoofing. The same implementation will continue to record sensor data and optimize new layers (and the current position of the robot) with respect to the existing layers.

**Figure 6:**
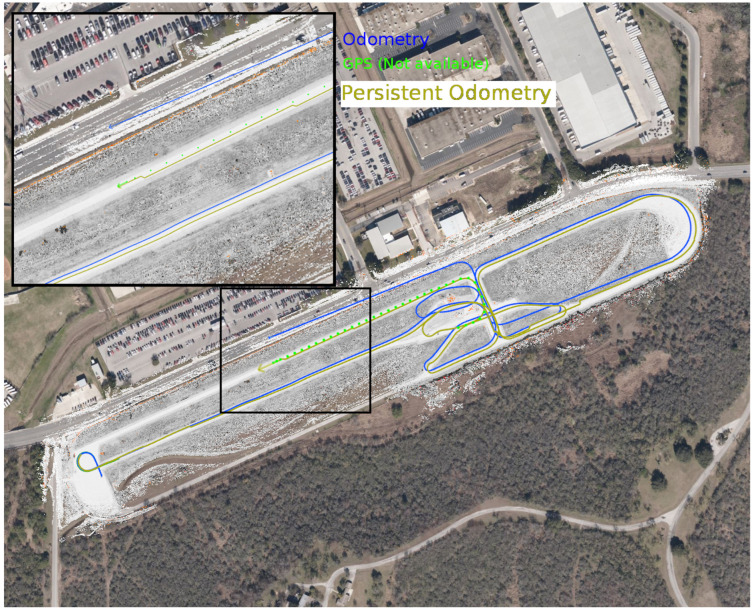
The result of localizing to an evolving map. This shows the result of retraversing a previously explored area, and localizing to the persistent map. The localization track is 2.3 km long, ending in an olive arrow inside the region inset in the upper left of the figure. This arrow is approximately 1.0 m from the GPS location at this point in time, shown by a light green dot. The odometry track is also 2.3 km long, ending in a blue arrow in the main road bordering the test track, approximately 32.8 m north and slightly west of the current GPS location. The satellite imagery and GPS coordinates (green dots) are shown only for context, and are not available to the system.

In this result, the test track map created in Section 6.1 is used to localize the vehicle in the absence of GPS. The GPS signal is blocked after about 10 m (dark green line in Figure 6), and the map is used to localize the vehicle as it traverses about 2.3 km. At this distance, after several loops and curves, odometry is off by 32.8 m. However, the map-based localization is still accurate to within 1 m. The GPS signals (shown in bright green) are used as a ground truth and visualization, though they are not available to the system.

**Figure 7:**
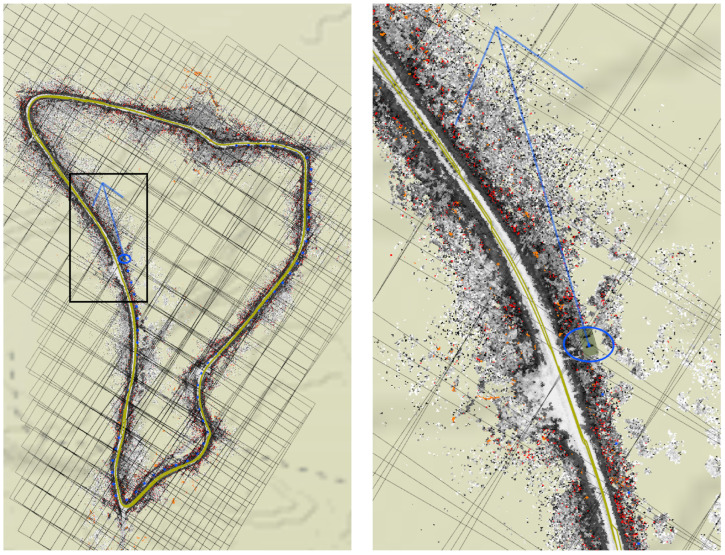
Localizing to an evolving map in off-road conditions. Here, the map from Figure 5 (on the left) is used to localize the vehicle even as it is evolves. The blue dots around the trail indicate the derived localization of the vehicle, with the blue ellipse showing the covariance of the estimate. The blue arrow indicates direction. The scattered dots off-trail are costmap data, including black-and-white for valid data, red for lethal obstacles, and orange for missing data. The map on the right, comprising the boxed area around the vehicle, show a zoomed-in view of the vehicle and its immediate surroundings.

Similarly, a map of a 1.2 km trail is created using GPS-available data, then used to localize a vehicle running without GPS for one and a half laps. After about 1.5 km of travel without GPS, the odometry is 11.5 m off-track. The trail at this point is about 3.8 m wide. The localization error with respect to the map is less than a meter, as shown in Figure 7.

### 6.4. Localization Based on a Static Persistent Map

There are many situations in which an operator could evaluate a collected persistent map and decide to stop evolving the map as new information is gathered. New information added to an area always has the potential, especially in the absence of GPS, to be mis-registered with existing data, leading to map corruption. As new manifolds are added to existing data, the optimization process described above generates extremely large datasets and becomes computationally unsustainable for missions lasting hours or days. Finally, human review of a derived map may be desired in safety-critical scenarios.

For any or all of these reasons, a new localization mode for the Persistent Map, called costmap-based localization, (CBL) has been developed in which the module suspends the accumulation and optimization of the map after an initial recording period and freezes the map for use as reference.

During CBL, the area of the map around the estimated location of the vehicle is periodically rendered into a persistent costmap. This persistent costmap is then matched via phase-correlation [44,46] with the most-recent real-time sensor-derived costmap to generate an estimated map-space pose of the vehicle. The usage of aggregated costmaps instead of obstacle-maps (also known as occupancy grids) or point clouds, common in other map-based localization algorithms, results in a robust matching process that can function even in areas without foliage or other vertical structures to align.

**Figure 8:**
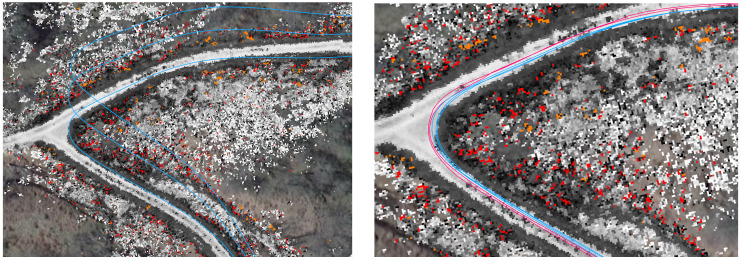
The same manually driven path is analyzed with and without reference to the persistent map. On the left, the vehicle’s state estimate, shown in blue, exhibits the expected odometric drift across repeated traversals of the same trail. On the right, costmap based localization, shown in red, prevents the vehicle’s state estimate, shown in blue, from drifting without GPS.

This pose is fused into the vehicle state estimate with appropriate covariances, both improving localization consistency while GPS is available and allowing for long-term GPS-independent operation in previously mapped areas, as illustrated in Figure 8. In the case that the original map was produced with GPS, the fused state estimate is accurate with respect to geolocated coordinates. Otherwise, the fused state estimate is still accurate with respect to the map, but both are warped with respect to geolocated coordinates. In both situations, the Persistent Map localization provides map-relative localization accuracy similar to or better than that of GPS. The Robotic Technology Kernel navigation suite is robust to this level of inaccuracy, and thus this enhancement allows these vehicles to operate normally in previously traversed areas even when GPS data are completely inaccessible.

**Figure 9:**
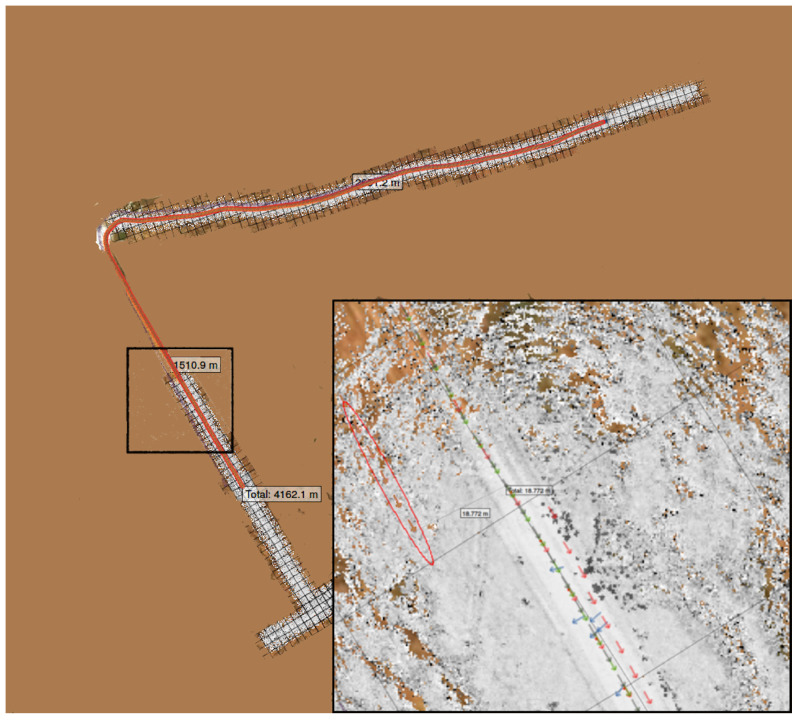
Costmap-Based Localization (CBL) on a static Persistent Map (part a). The (two part) persistent map, overlaid with four Kalman-filter based state estimates: GPS in green, GPS + CBL in red, dead reckoning in purple, and CBL only in brown. For scale, the map cells are about 25 m on a side, and the pictured path about 4.1 km. As expected, dead reckoning accumulated around 50 m of error in that time. The state estimates with GPS averaged under 2 m of error. The state estimator with no GPS but using costmap based localization stayed within 12 m for most of the route, drifting upwards between 250 and 425 s where there was no map to localize against (dead reckoning), then snapping back down to under 5 m when the vehicle re-entered the mapped region about 425 s into the route (see inset of left image, showing the 18 m jump when the map re-registered). The errors between these estimates are shown in Figure 10.

**Figure 10:**
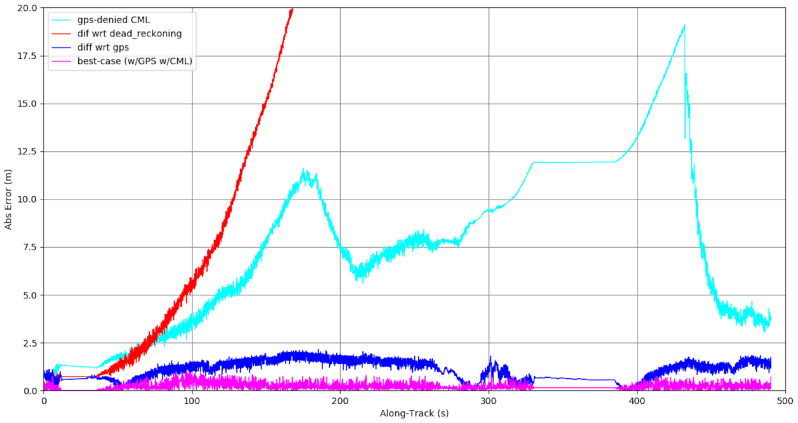
Costmap-Based Localization (CBL) on a static Persistent Map (part b). This graph shows the absolute error between several different Kalman-filter based localizations including state-of-the-art best case scenario (GPS, wheel encoders, and inertial measurements) in blue, dead reckoning (wheel encoders and inertial measurements) in red, and costmap based localization (CML, wheel encoders, and inertial measurements), in magenta with access to GPS or cyan without access to GPS, on the course shown in Figure 9.

Figure 9 and Figure 10 illustrate the benefit on this mode in extended missions. An approximately 4.5 km section of a sparse desert environment was mapped in two sections, with GPS available. On another day, the vehicle re-traversed this area without GPS. The figure shows the output of four Kalman-filter based state estimators: with and without GPS and with and without costmap based localization (CBL). Over the 4.1 km route, the dead reckoning (no GPS, no CBL) drifted ≈50 m from the best case position (with GPS, with CBL). Of particular interest to this paper, the GPS-denied localization (no GPS, with CBL) case tracked within 10 m of the best-case position during the first mapped leg, drifted up to 18 m in the unmapped region, and regained tracking when the map was re-entered at the end.

This shows GPS-denied performance in regions previously mapped with GPS at an accuracy comparable to GPS (about 8 m [47]).

**Figure 11:**
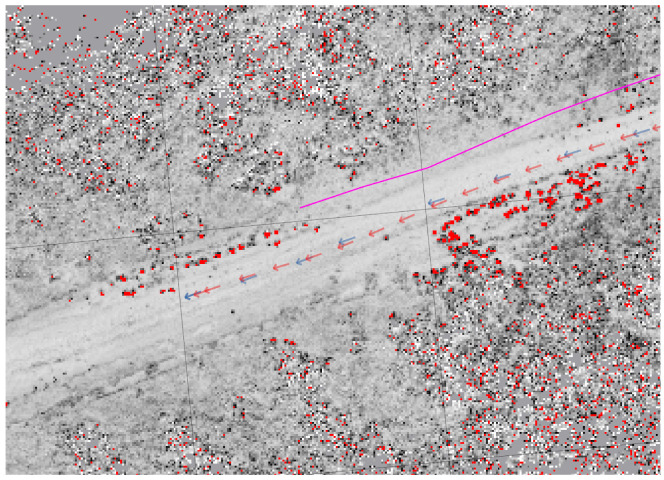
Costmap-Based Localization (CBL) on a static Persistent Map built without GPS. The GPS track, shown in magenta, is not available to the state estimator. The blue arrows indicate the localizations generated by the costmap based localization, and the red arrows show the Kalman filter-based state estimator that incorporates wheel encoders, inertial measurements, and gyroscope inputs with the localization results.

Finally, we show the results of generating a persistent map in the absence of GPS (The current implementation requires at least one GPS fix to register the global WGS84 coordinates with the local map coordinates. This could be replaced by a manual initialization), and then using that map to localize a vehicle in GPS-denied condition. Figure 11 shows that, while the persistent map generated is stretched and warped with respect to ground truth (observe the road, for example in the satellite imagery and the persistent map), the localization correctly places the vehicle in the center of the road in the persistent map. In this experiment, a vehicle drove in a desert environment for ≈3.2 km without GPS. By the western end of the track, the dead reckoning state estimator had drifted by ≈30 m, more than enough to cause the estimated vehicle to be completely off the wide road, and to miss the planned turn. While the generated map is off from the actual geo-located positions of the features, they are locally consistent and useful for navigation and planning. Thus, when the vehicle re-traverses this same area on a different day, again without the benefit of GPS, CML is able to localize the vehicle to a sufficient precision to allow automated route following.

### 6.5. Planning on the Persistent Map

**Figure 12:**
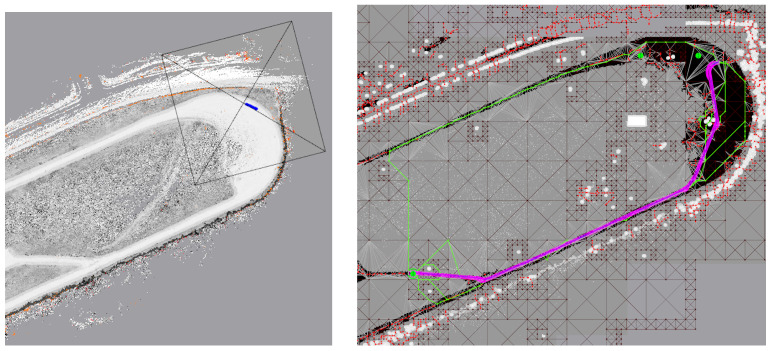
The persistent map may be used for path planning outside the current sensor range of the vehicle. On the (**left**), a portion of the persistent map seen in Figure 3, with the approximate current sensor footprint shown. On the (**right**), the averick Planner [34] is used to plan a path on this map. The planned path is visible in purple. The green and red lines show details of the path planning data structure.

Aggregating and saving sensor data as the vehicle moves allows consideration of paths and obstacles outside the current sensor range of the vehicle. As an example, Figure 12 shows the results of using the Maverick Planner [34] and the persistent map seen in Figure 3, to plan a 265 m path to an endpoint 225 m away that is not visible in current sensor imagery.

## 7. Conclusions

This paper presented the overall approach and some results from the persistent map, a module developed at Southwest Research Institute with funding from the Office of Naval Research and the United States Army Combat Capability Development Command Ground Vehicle Systems Center.

The persistent map separates acquired sensor-derived data into manifolds, then optimizes the placement of these manifolds into odometry space by way of a nonlinear minimization with cost terms representing:

- minimal deformation of source tiles;
- good alignment of any GPS fixes that are available;
- minimal fixed steady state error over each GPS source; and
- good alignment of data for overlapping costmaps.

The persistent map module allows limited vehicle operation in regions that have not been previously mapped, and may or may not have GPS coverage during a mission. This approach differs from traditional SLAM (Simultaneous Localization and Mapping) approaches in several important ways.

The first is the attention to local consistency of the map. In our use case, gapping or tearing of the sensor data can easily block paths (in the map) that are traversable in the world. The method of registering source tiles to vertices, then adjusting those vertices, mitigates this risk.

The second difference is the leveraging of GPS data when it is available, to allow operations in environments where GPS is prone to dropouts. The persistent map will use the GPS that is available, and when it is not the other cost terms will enforce the optimal configuration of the map.

The third difference is the use of costmaps, rather than raw sensor data, to help optimize the stretching of the manifolds. Other GVSC and SwRI projects have focused on processing sensor data to robustly identify paths, traversable areas, and lethal obstacles in the sensor data, and to represent them inside costmaps. These maps are then used to help guide the optimization process.

Finally, two applications of the persistent map are presented: planning and execution of paths well outside the current sensor distance of the vehicle, and localization of the vehicle across kilometers of GPS-denied travel, using a persistent map.
